# Fucoidan Extracted From Sporophyll of *Undaria pinnatifida* Grown in Weihai, China – Chemical Composition and Comparison of Antioxidant Activity of Different Molecular Weight Fractions

**DOI:** 10.3389/fnut.2021.636930

**Published:** 2021-05-28

**Authors:** Jing Yu, Qianqian Li, Jun Wu, Xiaotong Yang, Shiping Yang, Wei Zhu, Yang Liu, Wei Tang, Shaoping Nie, Amira Hassouna, William Lindsey White, Yu Zhao, Jun Lu

**Affiliations:** ^1^College of Life Sciences, Shanghai Normal University, Shanghai, China; ^2^College of Chemistry and Materials Science, Shanghai Normal University, Shanghai, China; ^3^Shanghai Institute of Quality Inspection and Technical Research, Shanghai, China; ^4^State Key Laboratory of Food Science and Technology, Nanchang University, Nanchang, China; ^5^Faculty of Health and Environmental Sciences, School of Public Health and Interdisciplinary Studies, Auckland University of Technology, Auckland, New Zealand; ^6^Department of Medical Biochemistry and Molecular Biology, Faculty of Medicine, Cairo University, Cairo, Egypt; ^7^School of Science, Faculty of Health and Environmental Sciences, Auckland University of Technology, Auckland, New Zealand; ^8^Institute of Biomedical Technology, Auckland University of Technology, Auckland, New Zealand; ^9^Maurice Wilkins Centre for Molecular Discovery, Auckland, New Zealand; ^10^College of Life Sciences and Oceanography, Shenzhen University, Shenzhen, China; ^11^College of Food Engineering and Nutrition Sciences, Shaanxi Normal University, Shaanxi, China

**Keywords:** fucoidan, molecular weight, antioxidant, fraction, chemical composition, nutraceutical

## Abstract

Fucoidan is a multifunctional marine carbohydrate polymer that differs in its chemical composition and bioactivity both between seaweed species and within species from different locations across the globe. In this study, fucoidan was extracted from the sporophyll of *Undaria pinnatifida* grown in Weihai, Shandong Province, China. Fucoidan fractions with molecular weight cutoffs (MWCO) of >300 kDa and <10 kDa were obtained via dialysis. The fucoidan standard from Sigma (Fstd, ≥95, CAS: 9072-19-9), fucoidan crude extract (WH), >300 kDa fraction (300k) and <10 kDa fraction (10k) were compared in terms of chemical composition and antioxidant capacity. Based on Fourier transform infrared spectroscopy (FT-IR) analysis, Fstd, WH, and 300k all showed strong bands around 830 cm^−1^, corresponding to the sulfate substituent in the molecule. The results showed that compared with WH and 300 k, the degree of sulfation at 10k was the lowest. From Nuclear magnetic resonance spectroscopy (NMR) result, the four fucoidan samples all contain α-_L_-fucose. The primary antioxidant ability of the 10k is significantly higher than that of the 300k, WH, and Fstd, but the secondary antioxidant capabilities of the 10k and 300k were similar, and both were higher than that of the butylated hydroxyanisole (BHA). The ferric reducing antioxidant ability was higher in the 300k and WH fractions. This demonstrates that fucoidan extracted from *U. pinnatifida* grown in Weihai, China should be a useful nutraceutical resource.

## Introduction

Oxidative stress is a condition of imbalance between the intracellular reactive oxygen species (ROS, produced during the aerobic metabolism) production, and scavenging by the antioxidant defense system (1). An excess of ROS has a damaging effect on cellular organelles as well as biomacromolecules, including proteins, lipids, and nucleic acids (2). The increased intracellular damage eventually leads to cell dysfunction and various diseases may occur (3). Discovering non-toxic compounds with strong antioxidant activity is beneficial for the prevention and treatment of diseases caused by oxidative stress. Hence there is increased research in isolating bioactive compounds from terrestrial and marine organisms (1). Seaweeds contain high amounts of polysaccharides with many bioactivities, including antioxidant, anti-inflammation, anti-diabetes, anti-coagulant, anti-hypertension, and anti-cancer activities.

Fucoidan is an antioxidant (4), which is widely available *from* cheap sources compared to other polysaccharides (5). According to reports, fucoidan usually has strong secondary antioxidant activity, comparable to synthetic antioxidants such as butylated hydroxyanisole (BHA) and butylated hydroxytoluene (BHT), which are known to have adverse effects on humans. Therefore, fucoidan has become a focus in functional food research in recent years (6).

Fucoidan is a cell wall polysaccharide normally composed of fucose, uronic acid, galactose, xylose and sulfate (7), which can be obtained from some brown seaweeds (8). The structure of fucoidan is extremely complex and generally falls into one of two types. One has repetitive (1 → 3)- l-fucopyranose and the other has alternate repetitive (1 → 3)- and (1 → 4)- l-fucopyranose (9). Changes in uronic acid and sulfate contents can create different antioxidant activities (4). The antioxidant and anticoagulant activities of polysaccharides are also strongly dependent on the degree of sulfation. Studies have shown that the use of different extraction processes can cause differences in its antioxidant properties (10). Moreover, the sulfate content of fucoidan can differ seasonally within one location (7).

In this research, fucoidan was extracted from sporophyll of *U. pinnatifida* grown in the Weihai sea region, and dialysis was used to obtain different molecular weight fractions of fucoidan. The chemical composition and antioxidant capacities of crude fucoidan and its fractions were studied and compared.

## Materials and Methods

### Materials

Fucoidan standard extracted from *U. pinnatifida* (Fstd) was purchased from Sigma-Aldrich (Buchs, Switzerland). Sugar standards including _L_-fucose, _D_-galactose, and _D_-glucose were purchased from Macklin (Shanghai, China). Synthetic antioxidants BHA, sodium hydroxide (≥99.0%), and ascorbic acid were also purchased from Macklin (Shanghai, China). Anhydrous ethanol (≥99.0%), hydrochloric acid (4 M), trifluoroacetic acid (≥99.0%), ferrous sulfate (9 mM), hydrogen peroxide (0.3%), ethanolic solution of salicylic acid (5 mM), potassium ferricyanide, 1,1-diphenyl-2-picrylhydrazyl radical 2,2-diphenyl-hydrazyl (DPPH) and dialysis bags were purchased from Solarbio (Beijing, China). All other chemicals used in this study were of analytical grade.

### Extraction of Fucoidan

The brown seaweed species *U. pinnatifida* is native to Weihai, Shandong, China. Harvest took place in June 2019. Extraction was carried out according to a previously published method (11). In brief, the fresh *U. pinnatifida* was oven-dried to constant weight (40°C, DHG-9070A-Type, Yiheng Corporation, Shanghai China) and ground to powder, and then stored at room temperature until use. The powder was extracted in water at 70°C overnight. After centrifugation (Medical Centrifuge, H2050R, Xiangyi Corporation, Changsha, Hunan, China), 2% CaCl_2_ powder was added, kept at 4°C overnight, and the alginate was removed by centrifugation. Anhydrous ethanol was added to the supernatant to a final concentration of 70% ethanol. Fucoidan precipitated by ethanol was collected by centrifugation, and freeze-dried (VirTis 2KBTes-55 Freeze Dryer, SP Scientific, Warminster, PA, USA) to obtain crude fucoidan powder, which was stored at 4°C until used. Subsequently, dialysis was performed to obtain fucoidan with different molecular weight fractions. Crude fucoidan of 1.5 g was dissolved in 50 mL of water. The solutions were placed in 300 kDa and 10 kDa molecular weight cut-off dialysis bags and kept at 4°C for 3 days without changing water. The retentate of 300 kDa bag was collected and freeze-dried to obtain > 300 kDa fraction powder. The dialysate of the 10 kDa bag was collected, concentrated by rotary evaporation, and freeze-dried to obtain <10 kDa fucoidan fraction powder. The low molecular weight fraction might contain other small carbohydrate molecules such as oligosaccharide and phenolic compounds, which were unable to be separated out due to the current procedure design.

### Determination of Total Sugar Content

The content of total sugar in fucoidan was determined according to a previously described method (12). Standard fucose of 10 mg was dissolved in 250 mL water. Volumes of 0.4, 0.6, 0.8, 1.0, 1.2, 1.4, 1.6, 1.8 mL were drawn, and made up to 2 mL with distilled water. Then, 5% 1 mL of phenol and 5 mL of concentrated sulfuric acid were added the same as the samples. Standard solutions were shaken, cooled, left at room temperature for 20 min, and the absorbance was measured at 490 nm to construct a standard curve. 2 mL each of Fstd, WH, 300k, and 10k samples were prepared, and 1 mL of 5% phenol and 5 mL of concentrated sulfuric acid were added. The solution was shaken, cooled, left at room temperature for 20 min, when absorbance was measured at 490 nm using UV-Vis spectrometer (General Analysis Instrument Corporation, Beijing, China).

### The Molecular Weight Distribution

The molecular weight distribution of the fucoidan was measured by high-performance gel permeation chromatography (HPGPC) according to a previously published method 16. An Agilent 1260 HPLC system (Agilent, Santa Clara, CA, USA) was used, including a refractive index detector (RID, DEAA602884), a variable wavelength detector (VWD, DEABB05037), and an Ultrahydrogel^TM^ linear column (7.8 × 300 mm) (Waters, Milford, Massachusetts, USA). The column was maintained at 35 ± 0.1°C. The mobile phase was NaNO_3_/0.02% NaN_3_ (0.1 M), and the flow rate was 0.6 mL/min.

### Determination of Monosaccharide Content

High Performance Anion Exchange Chromatography (HPAEC) is coupled with a Pulsed Amperometric Detector (HPAEC-PAD) (Thermo Fisher Scientific, Waltham, MA, USA) for the separation and determination of monosaccharide constituents of polysaccharides (13). Each fucoidan sample (5 mg) was hydrolyzed in sulfuric acid (12 M, 0.5 mL) for half an hour. HPAEC-PAD analysis was performed using a Dionex ICS-2500 system (Dionex Corporation, Sunnyvale, California, USA) equipped with a CarboPac™ PA20 analytical column (250 mm x 4 mm ID, Dionex Corp., Sunnyvale, CA, USA) and CarboPac™ PA20 guard column (50 mm × 4 mm ID, Dionex). For the mobile phase, CH_3_COONa (1 M), H_2_O, and NaOH (250 mM) were used. The injection temperature was maintained at 30°C.

### Determination of Sulfate Content

Sulfate content was analyzed using the barium chloride–gelatin method (14). Briefly, gelatin (2 g) was dissolved in 400 mL of deionized water to prepare a gelatinous fluid. Two grams of barium chloride were dissolved in a gel-like fluid to prepare a BaCl_2_-gelatin reagent. Fucoidan samples (WH, 300k, 10k, and Fstd) were prepared by dissolving 10 mg of the sample in 5 mL of 4 M HCl, respectively. Each sample was allowed to hydrolyze at 90°C for 2.5 h. The hydrolyzed samples (0.1 mL) were pipetted into 15 mL centrifuge tubes, to which 1.9 mL of 3% TCA and 0.5 mL of BaCl_2_-gelatin reagent were added. The reaction mixture was vortexed and transferred into a cuvette, and the absorbance was measured at 360 nm. The analysis was performed in triplicates. Potassium sulfate solution was used to construct a standard calibration curve.

### Determination of Uronic Acid Content

A previously described method (15) was used to determine the uronic acid content of the fucoidan samples. Samples were heated in a boiling water bath for 10 min, and then quickly cooled. Carbazole-anhydrous ethanol of 0.2 mL was added, the sample placed in a boiling water bath, cooled to room temperature, and the absorbance measured at 530 nm. All analyses was performed in triplicate.

### Fourier Transform Infrared Spectroscopy (FTIR)

Fourier transform infrared spectroscopy (FTIR) was used to obtain infrared spectra of samples using a Thermo Scientific Nicolet 380 FTIR spectrometer (Thermo Fisher Scientific, Waltham, MA, USA). All fucoidan and its fraction samples (Fstd, WH, 300k, 10k) were ground into powders, and the fucoidan and KBr powders were ground, mixed and pressed into flakes at a ratio of 1: 100 for FTIR analysis. The software used was Origin 7.0 (OriginLab, Northampton, MA, USA). Sample spectra were collected between 400 and 4000 cm^−1^ at a resolution of 4 cm^−1^. The spectrum was then analyzed to determine possible bond types, and functional groups.

### Nuclear Magnetic Resonance Spectroscopy (NMR)

10 mg WH, 300k, 10k were prepared by exchanging lyophilized material twice with D_2_O, followed by dissolving in 1.0 mL 99.99% D_2_O. NMR spectra (^1^H NMR) were recorded on Advance II 400 MHz spectrometer (Bruker, Switzerland).

### Determination of Antioxidant Capacity of Fucoidan

#### Free Radical Scavenging Assay (DPPH)

The DPPH free radical scavenger of the fucoidan was determined using a modification of the method described by (7). A DPPH working solution was prepared immediately before the experiment by dissolving 40 mg of DPPH in a 1 L ethanol solution. This was stored in the dark at room temperature until analysis. Fucoidan samples (Fstd, WH, 300 k, and 10 k) were prepared by dissolving 2 mg of sample in 1 mL of deionized water. Three mL of fucoidan sample was added to 3 mL of DPPH working solution, vortexed, kept for 40 min at room temperature, protected from light, and then absorbance was measured at 517 nm (The molar concentrations of WH, 300 k and 10 k are 3.74 × 10^−5^ mol/L, 1.28 × 10^−6^ mol/L, 2.07 × 10^−3^ mol/L, respectively). The analysis was performed in triplicates each time. The free radical scavenging activity of the fucoidan sample was determined according to the equation:

$$\begin{array}{l} \%\text{ inhibition}=\left[\left({\text{A}}_{0}-{\text{A}}_{1}\right)/{\text{A}}_{0}\right]\times 100 \end{array}$$

where A_0_ = Abs_517_ of control (without sample), A_1_ = Abs_517_ of sample.

#### Hydroxyl Radical Scavenging Assay (·OH)

The hydroxyl radical scavenging activity of fucoidan samples was determined using the below described method. Hydroxyl radicals were generated by the Fenton reaction and detected by their ability to hydroxylate salicylate. The reaction contained fucoidan solution (0.1 mL, 1 mg/mL), ferrous sulfate (9 mM, 1 mL), hydrogen peroxide (0.3%, 1 mL) and ethanolic solution of salicylic acid (5 mM, 0.5 mL). It was followed by incubation at 37°C for 30 min. The final volume was completed to 5 mL using distilled water and the absorbance was recorded at 510 nm (The molar concentrations of WH, 300 k, and 10 k are 1.87 × 10^−5^ mol/L, 0.64 × 10^−6^ mol/L, 1.04 × 10^−3^ mol/L, respectively). The hydroxyl radical scavenging activity of ascorbic acid and BHA (2 mg/mL) was also determined using the same method. The analyses were conducted in triplicate. The hydroxyl radical scavenging activity of the samples was determined according to the following equation:

$$\begin{array}{l} \text{O}{\text{H}}^{-}\text{radical scavenging rate }\left(\text{\%}\right)=\left\{1-\left[\left({\text{A}}_{1}-{\text{A}}_{2}\right)/{\text{A}}_{0}\right]\right\}\times 100 \end{array}$$

Where A_0_ = Abs_510_ of control (without sample), A_1_ = Abs_510_ of reaction containing sample and salicylic acid, A_2_ = Abs_510_ of reaction containing sample without salicylic acid.

#### Determination of Reducing Power

The reducing power analysis was performed according to a previously described method (16) with slight modifications. Potassium phosphate buffer (0.1 M, 2.5 mL, pH 6.6) and 1% potassium ferricyanide (2.5 mL) were mixed with 1 mL of different dilutions. The reaction mixture was incubated at 50°C for 20 min, and then 10% of trichloroacetic acid (2.5 mL) was added. After that, 2.5 mL of water and 0.5 mL of 0.1% FeCl_3_ were added to 2.5 mL of the reaction mixture. The solution was incubated at ambient temperature (25°C) for 30 min to develop color. The absorbance was then measured at 700 nm.

### Statistical Analysis

All the data were presented as means ± SD. A *P*-value <0.05 was considered statistically significant after passing Duncan's multiple-range test. Data were processed using SPSS (v.17.0, IBM, Armonk, NY, USA).

## Results and Discussions

### Determination of Total Sugar Content

**Figure 1:**
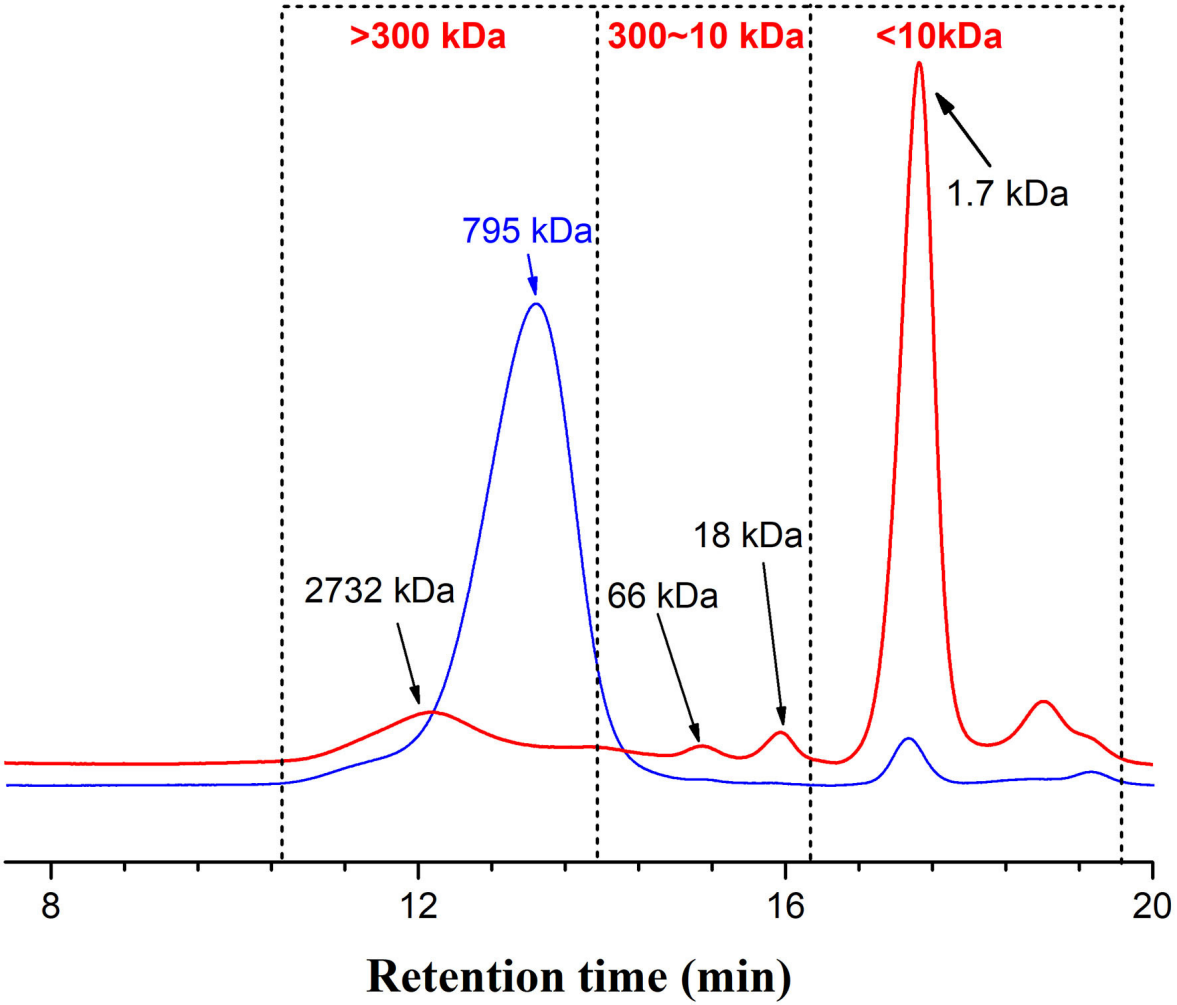
Profiles of Fstd (blue line) and WH (red line), monitored by high-performance gel permeation chromatography (HPGPC), coupled with a refractive index detector (RID). Fstd, fucoidan standard; WH, fucoidan extracted from Weihai seaweed *Undaria pinnatifida*.

A dialysis bag was used to separate fucoidan into different molecular fractions, which recovered 90% of the fucoidan, with 33% of the recovered fraction <10 kDa and over 60% of the fraction > 300 kDa. However, the peak area of >300kDa for WH in HPGPC (Figure 1) is apparently inconsistent with the fraction percentage data, which may be caused by disturbance of absorption by certain impurities. Polysaccharides were first hydrolyzed into monosaccharides under the action of concentrated sulfuric acid and quickly dehydrated to form aldehyde derivatives, and then to form orange-yellow compounds with phenol. The total sugar content of all fucoidan samples is summarized in Table 1. The >300kDa fraction (300k) has the highest total sugar content, whereas the fraction <10 kDa (10k) has the lowest total sugar content. Since the fraction between 10 and 300 kDa only accounts for <10% of the fucoidan crude extract (WH), the total sugar content and other biochemical analyses were not pursued due to the limited availability of the material. The low percentage of total sugar in 10k was due to high metal ion content, where the ash content accounted for ~40% of the total weight.

**Table 1:** Chemical components of each fucoidan sample.

**Sample**	**Sugar Content (% w/w)**	**Sulfate Content (% w/w)**	**Uronic Acid Content (% w/w)**
Fstd	52.30% ± 0.02^a^	41.33% ± 0.15^a^	4.741% ± 0.01^b^
WH	48.64% ± 0.03^c^	37.39% ± 0.03^b^	8.83% ± 0.01^a^
300k	51.87% ± 0.02^b^	39.44% ± 0.03^b^	4.27% ± 0.01^b, c^
10k	25.77% ± 0.03^d^	25.88% ± 0.02^c^	2.98% ± 0.01^c^

*Fstd = fucoidan standard (≥95%, CAS: 9072-19-9). WH = fucoidan crude extract. 300k = fucoidan fraction with molecular weight over 300 kDa. 10k = fucoidan fraction with molecular weight <10 kDa. Values are expressed as mean with standard deviation (n = 3). Within each column, mean values with different superscript lowercase letters are statistically different (p <0.05)*.

### The Molecular Weight Distribution

The molecular weight (MW) of fucoidan is one of the important parameters affecting its biological activity (12). It has been suggested that the low MW fraction of fucoidan has higher antioxidant activity than the high MW fraction of fucoidan. Our high-performance gel permeation chromatography (HPGPC) results (Figure 1) indicate that each fucoidan sample contains a big peak of high MW fraction and a small peak of low MW fraction. The elution curve of fucoidan standard from Sigma (Fstd) shows that the major large MW fraction is about 2500 kDa, the minor small MW fraction exceeds 2 kDa, and a small fraction is also present below 1 kDa. It also contains a small peak at ~273.1 kDa and a peak at ~1.7 kDa. The elution curve of WH shows that it contains a large fraction with MW between 209 and 5200 kDa, with a large peak at ~794 kDa and a small peak at ~2.1 kDa. The high peak of small molecular weight fraction may be a result of the existence of oligomeric molecules which may contain phenolic compounds.

### Determination of Monosaccharide Content

**Figure 2:**
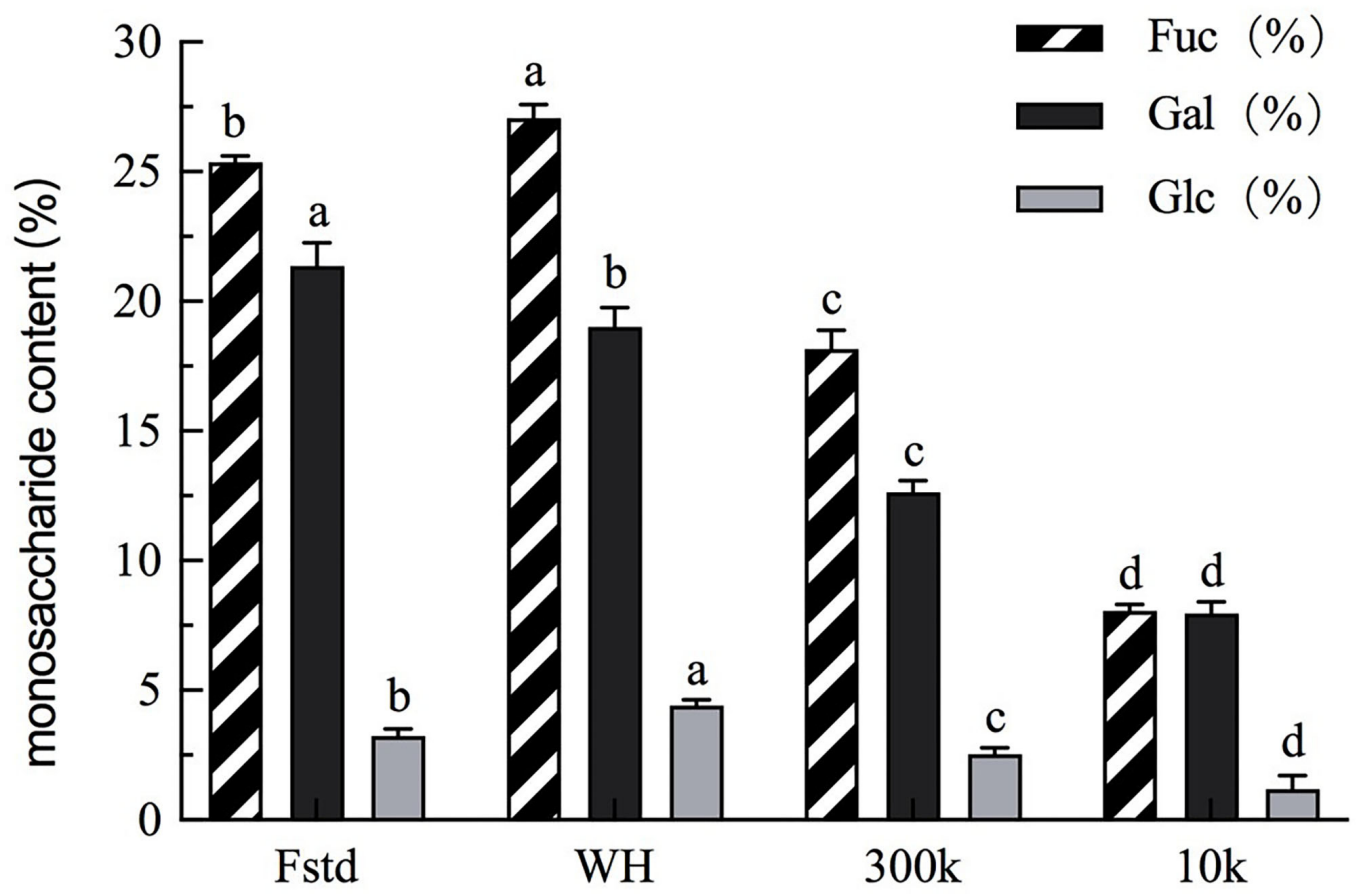
Monosaccharide content of each fucoidan sample. Fstd = fucoidan standard (≥ 95%, CAS: 9072-19-9). WH = fucoidan crude extract. 300k = fucoidan fraction with molecular weight over 300 kDa. 10k = fucoidan fraction with molecular weight <10 kDa. Values are expressed as mean with standard deviation (*n* = 3). Within each column, mean values with different superscript lowercase letters are statistically different (*p* < 0.05).

The monosaccharide composition of fucoidan crude extract, fucoidan standard, 300k, and 10k was analyzed by HPLC. Fucose is a characteristic neutral sugar for all fucose polysaccharides (17). In the four fucose polysaccharide samples, three major monosaccharides were identified: fucose, galactose and glucose (Figure 2). Minor monosaccharides included xylose, mannose and rhamnose. The analysis of those minor monosaccharides was not pursuit. The most abundant monosaccharides in common fucoidan are fucose, and galactose (18). The ratio of fucose to galactose is nearly 1:1 by calculation which is similar to those reported in the literature (19). The fucose content of fucoidan crude extract (WH) from Weihai *U. pinnatififida* is the highest among all fucoidan samples, and even higher than the fucose content of the fucoidan standard (Fstd). In previous papers, fucoidan with effective anti-tumor activity has a higher percentage of full-branched chains than other Fucoidan extracts that do not exhibit this biological activity (in 3- *O* and 4- *O* position) (20). There is a slight discrepancy between the sum of the monosaccharides in Figure 2 and the total sugar amount in Table 1, particularly in 300k and 10k fractions. This indicates that some alginate and its breakdown fractions, as well as other oligosaccharides may exist.

### Determination of Sulfate Content

Fucoidan has shown anti-cancer activity (21), and the sulfation level has been identified to be a key factor (22). Excessive sulfation causes the molecule to have a high negative charge, so that the fucoidan protein complex can be formed. This may hinder cell proliferation, thereby indirectly exerting biological activities such as antioxidants. In a recent study, Cho et al. (23) has reported that persulfate fucoidan has better α-amylase inhibition than native fucoidan. In addition, the position of the sulfate groups on the fucoidan may affect its biological activity (24). The barium chloride gelatin method is used to determine the sulfate content of Fstd, WH, 300k and 10k. As shown in Table 1, the sulfate content of Fstd is higher than that of WH. The sulfate content in fucoidan is affected by many factors, the main factors including type, harvest season and extraction method (25). January et al. compared the effects of different extraction methods on the sulfate content of fucoidan isolated from *U. pinnatifida* (26). The fucoidan obtained by the acid extraction method has a high sulfate content. The results of this article are consistent with this view. In addition, the sulfate content of 10k is lower than that of 300k. Bittkau et al. (27) separated fucoidan into several molecular weights, indicating that high-molecular-weight fucoidan contains higher sulfates than low-molecular-weight fucoidan.

### Determination of Uronic Acid Content

Fucoidan has the ability to regulate blood lipids, and the uronic acid content plays an important role in this regulation (28). Table 1 shows the measurement results of the uronic acid content of Fstd, WH, 300k, and 10k. WH has the highest uronic acid content (8.83% ± 0.01). Koh et al. (8) studied fucoidan crude extract from New Zealand *U. pinnatifida* and proposed that most uronic acid residues are attached to the main chain. Our results show that in terms of uronic acid content, the order is WH>300k>10k. The difference between the results of Koh et al. and others may be related to the source of fucoidan. In addition, uronic acid may also come from other polysaccharides and/or oligosaccharides. Therefore, the amount may be influenced by extraction methods and fractionation procedures. Zhang and co-workers analyzed the physical and chemical components of fucoidan extracted from *Sargassum fusiforme* from different sources, and the content of uronic acid is different (29). Therefore, for Weihai fucoidan, the crude extract may be more effective in regulating blood lipids.

### Fourier Transform Infrared Spectroscopy (FTIR) Analysis

**Figure 3:**
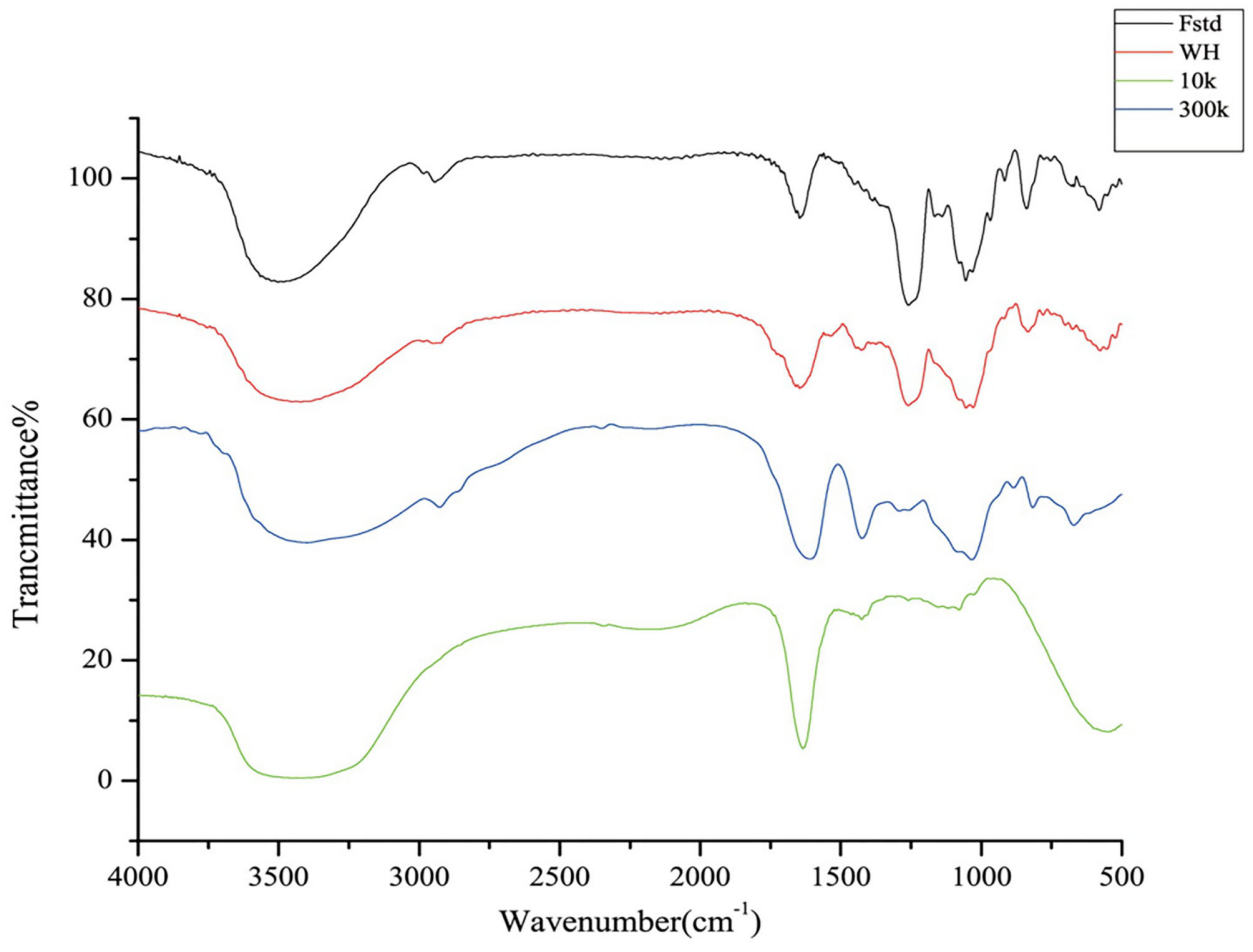
FTIR spectrum of four fucoidan samples. Fstd = fucoidan standard (≥ 95%, CAS: 9072-19-9). WH = fucoidan crude extract. 300k = fucoidan fraction with molecular weight over 300 kDa. 10k = fucoidan fraction with molecular weight <10 kDa.

FTIR analysis was performed to determine whether the sample had fucoidan characteristic infrared absorption. FTIR spectra of Fstd, WH, 300k, 10k show characteristic bands of fucoidan (Figure 3). All four fucose polysaccharides have stretching vibrational absorption peaks of hydroxyl groups near 3400 cm^−1^, which confirms the existence of -OH extension of fucose and galactose. Another weak signal was recorded at the region of 2930 cm^−1^ which indicated the presence of C-H stretching vibrations of the pyranoid ring and C6 groups of fucose (30), which was not as evident in 10k. There was an absorption peak at 1245 cm^−1^, which is considered to be the characteristic absorption peak of acetyl group. Meanwhile, the band of 1160-1170 cm^−1^ indicated the presence of S=O stretching of alkyl sulphoxide, which did not exist in 10k as well. Those at 1640 cm^−1^ indicated the presence of the C=O stretching vibration of O-acetyl groups (31). A strong band neared 830 cm^−1^ has been observed in Fstd, WH and 300k, which corresponds to the COS bending vibration of the sulfate substituent, indicating the existence of sulfate ester substituents. The lack of peak in the 1200-800 cm^−1^ region in 10k is an indication of low content of carbohydrates (8) which contains low molecular weight compounds such as phenolic compounds or pigments.

### Nuclear Magnetic Resonance Spectroscopy (NMR) Analysis

**Figure 4:**
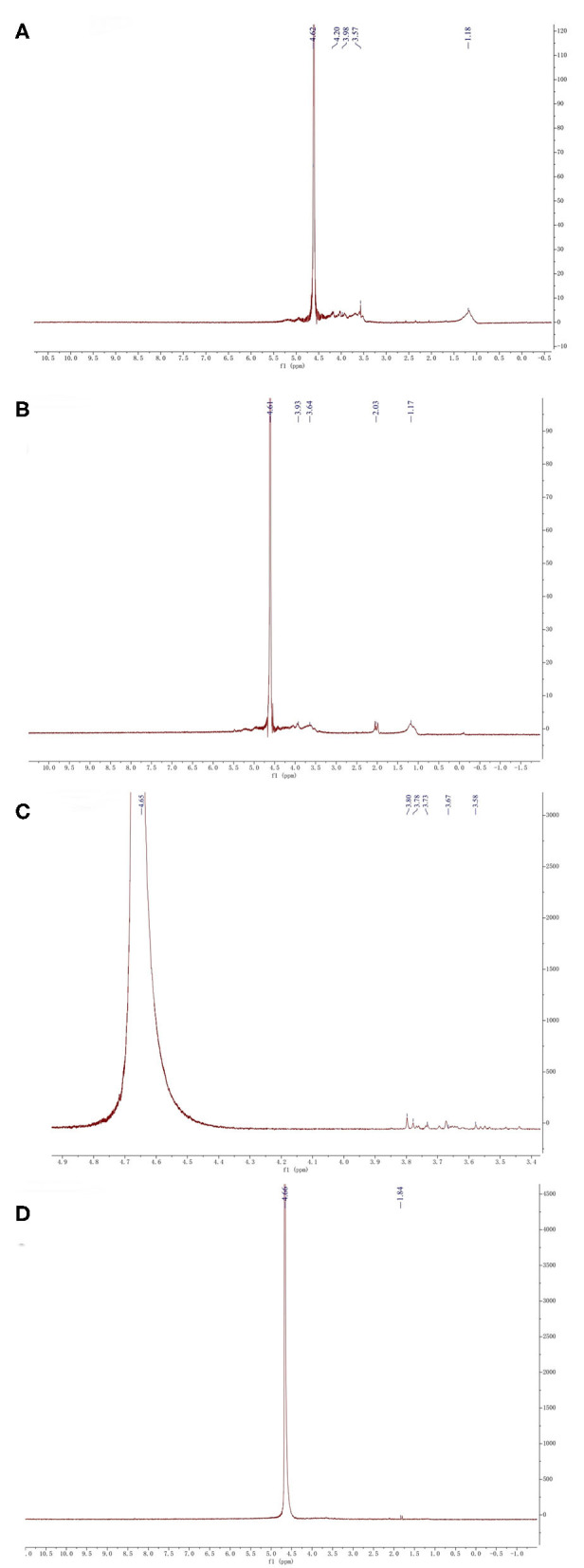
^1^HNMR analysis of four fucoidan samples. ^1^HNMR analysis of Fstd sample **(A)**, ^1^HNMR analysis of WH sample **(B)**, ^1^HNMR analysis of 300k sample **(C)**, ^1^HNMR analysis of 10k sample **(D)**.

NMR spectroscopy is an excellent tool to investigate the anomeric configuration and the sulfation pattern of the polysaccharides. The ^1^HNMR spectrum of fucoidan samples was displayed (Figure 4).

The signal at 4.61 corresponds to 3-linked α-_L_-fucose which is one of the main components of fucoidan (32). From the NMR results of the four fucoidan samples, 3-linked α-_L_-fucose was present in all four fucoidan samples. The signal at 3.92 represents 3-linked β-d-galactose, which was contained in Fstd and WH. The signal between 3.50 and 3.70 indicates the presence of α-_L_-rhamnosyl residues, the other three fucoidan samples contain it except for 10k. The signals at 2.03 indicates the existence of α carbon proton (33), which was contained in WH. What's more, signals between 1.10 and 1.40 which were assigned to the methyl protons (CH_3_) of fucopyranose residues, which was contained in Fstd merely. This may be related to the source of fucoidan.

**Figure 5:**
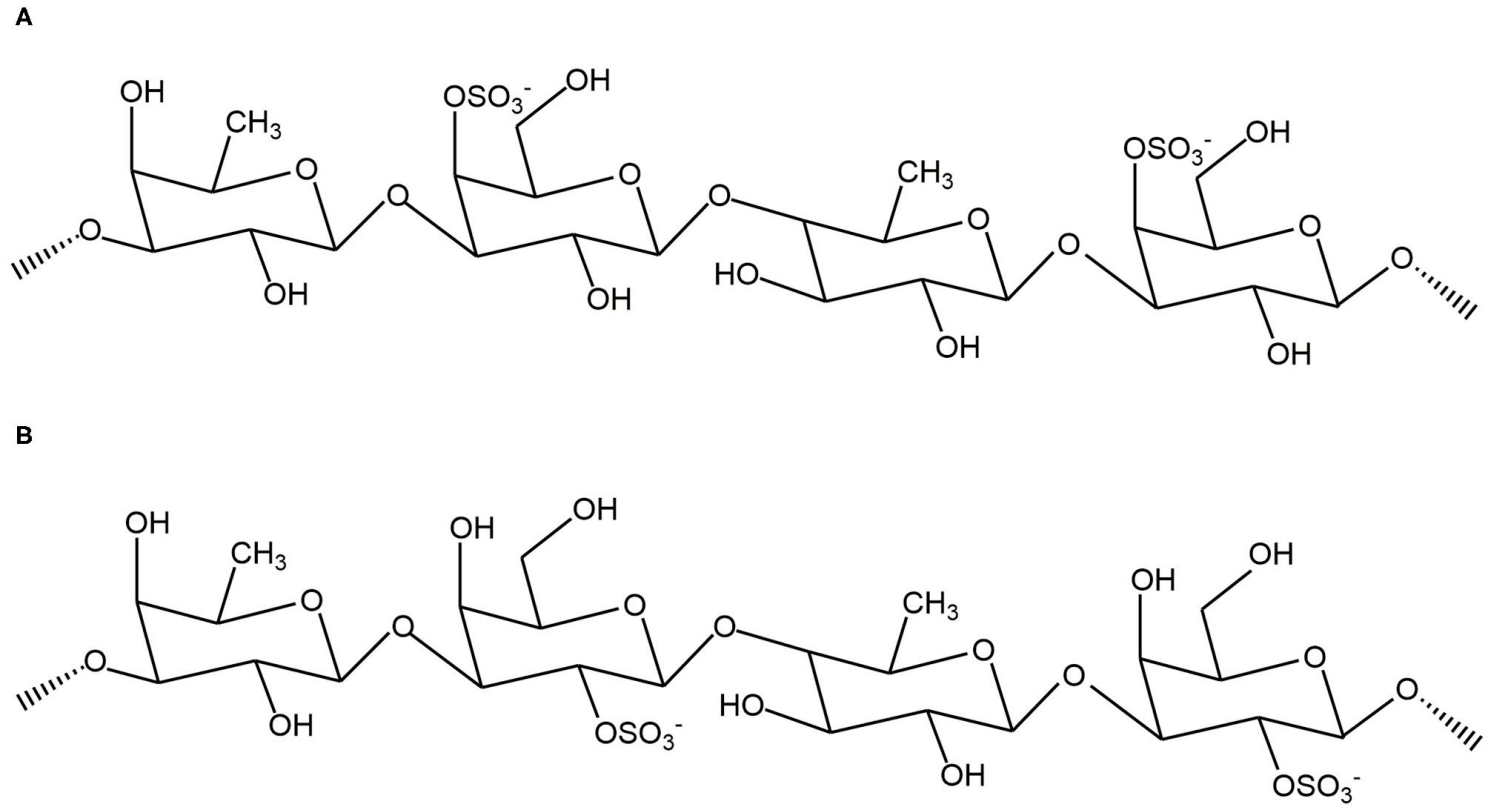
Proposed backbone structures of fucoidan crude extract with panel **(A)** showing C4 sulfation and panel **(B)** showing C2 sulfation.

Based on the results from the monosaccharide, FTIR, and NMR analyses, we confirmed that WH is composed of a fucose – galactose backbone linked via glycosidic bonds in an alternating (1 → 3)-_D_-galactose-(1 → 3)-_L_-fucose and (1 → 3)-_D_-galactose-(1 → 4)-_L_-fucose arrangement. Since the specific position of the sulfate group cannot be determined, we used previous studies which have shown that the sulfate group is likely to be at C4 or C2 position (4) to extrapolate the structure (Figure 5A showing C4 sulfation and Figure 5B showing C2 sulfation). Since there are still branches along the backbone, the fucose-galactose ratio is not strictly 1:1. Our analysis showed that fucose content slightly higher than the galactose content. This may indicate that more fucose molecules are present in the side chains and branches of the polysaccharide.

### Determination of Antioxidant Capacity of Fucoidan

#### Free Radical Scavenging Assay (DPPH)

The DPPH scavenging test was used to further study the antioxidant activity of Fstd, WH, 300k, and 10k. The 10k exhibited a higher level of antioxidant capacity than all other three fucoidan samples (Fstd, WH, 300k, Table 2), even though it contains less polysaccharide content. This may be caused by impurities in that fraction accumulated in the extraction and fractionation process. The impurities could be phenolic compounds, oligosaccharides and/or pigment molecules such as fucoxanthin, which have strong primary antioxidant activities (34). The scavenging effects of WH and 300k are similar, which indicates that most of the hydrogen atoms are not removed by the purification method. In general, the primary antioxidant capacities of the four fucoidan samples are significantly lower than that of BHA and ascorbic acid.

**Table 2:** Antioxidant activities and reducing power of Fstd, WH, 300k, 10k, ascorbic acid and BHA.

**Sample**	**DPPH Trolox Equivalent (μg/mL)**	**·OH Scavenging (%)**	**Reducing power (ug/mL)**
Fstd	204.05 ± 47.79^e^	30.50% ± 0.13^b^	4.20 ± 1.55^c^
WH	650.56 ± 187.55^d^	10.90% ± 0.08^c^	24.81 ± 2.07^a^
300 k	559.29 ± 216.44^d^	26.70% ± 0.09^b^	24.32 ± 0.57^a^
10 k	1822.15 ± 39.24^c^	26.60% ± 0.08^b^	11.36 ± 2.04^b^
BHA	4183.90 ± 78.20^b^	18.10% ± 0.02^bc^	
Ascorbic acid	4576.28 ± 5.23^a^	47.70% ± 0.01^a^	

*Fstd = fucoidan standard (≥95%, CAS: 9072-19-9). WH = fucoidan crude extract. 300k = fucoidan fraction with molecular weight over 300 kDa. 10k = fucoidan fraction with molecular weight <10 kDa. Values are expressed as mean with standard deviation (n = 3). Within each column, mean values with different superscript lowercase letters are statistically different (p <0.05)*.

#### Hydroxyl Radical Scavenging Assay (·OH)

Hydroxyl radicals cause damage to most biomolecules (35), including proteins in human cells and membranes, DNA, and polyunsaturated fatty acids (PUFA) (36). Therefore, the removal of hydroxyl free radicals is essential for the defense of antioxidants in cells (37).

Table 2 shows the scavenging results of hydroxyl radicals of four fucoidan samples (Fstd, WH, 300k, 10k), BHA and ascorbic acid. Compared with BHA, 300k, and 10k showed significantly higher hydroxyl radical scavenging activity. This result is consistent with the findings of (8). Hence, both high and low molecular weight fractions of fucoidan from Weihai can be used as effective hydroxyl radical scavengers. According to the literature, low molecular weight fucoidan shows higher hydroxyl radical scavenging activity. However, the results of this study show that the scavenging rate of hydroxyl radicals of fucoidan with low molecular weight (10k) was slightly lower than the high molecular weight fraction (300k). Similarly, the content of acid sugar (uronic acid) also plays an important role in the antioxidant properties of fucoidan (38). Demonstrated that the scavenging effect increased with increasing uronic acid content in different tea polysaccharide conjugate fractions. The presence of uronic acid may affect the physicochemical properties of the polysaccharide, and thus its biological activity. Our study shows that 10k fucoidan fraction has a uronic acid content less than that of 300k (4.27% ± 0.01). In addition (8), proposed that the sulfate groups in the low molecular weight polysaccharides responsible for the secondary antioxidant activity are more accessible than those in the high molecular weight fraction, resulting in significantly higher secondary antioxidant activity. Therefore, the hydroxyl radical scavenging effect of fucoidan is not only related to molecular weight, but also to the uronic acid content.

Studies suggest that fucoidans possess strong *in vitro* and *in vivo* antioxidant activities (39). The antioxidant activity of fucoidan is likely due to its ability to stimulate the expression of endogenous antioxidant enzymes. In a recent study (40), fucoidan with a molecular weight of 102.67 kDa (HFPS-F4) was isolated from *Hizikia fusiforme* (HFPS), a popular edible brown seaweed, to investigate its antioxidant activity. HFPS-F4 contains 99.01% fucoidan which consists of 71.79 carbohydrates and 27.22 sulfate content. It can increase the viability of H_2_O_2_-treated Vero cells by 5.41, 11.17, and 16.32% at the concentration of 12.5, 25, and 50 μg/mL, respectively. This is due to the induction of total nuclear factor (erythroid-derived 2)-like 2 (Nrf2) levels, which consequently leads to diminishing apoptosis by scavenging intracellular reactive oxygen species (ROS). In addition, the *in vivo* results show that the survival rates, ROS, and lipid peroxidation in H_2_O_2_-stimulated zebrafish have been improved with the fucoidan pretreatment (40).

#### Determination of Reducing Power

Our results are shown in Table 2. It is reported that there is a direct positive correlation between reducing power and antioxidant activity (41). In the reaction system, the antioxidant components in the sample cause the Fe ^3+^ /ferricyanide complex to reduce to Fe ^2+^, which can be monitored by measuring the formation of Prussian blue at 700 nm. The reducing power of WH and 300k is higher than 10k. In previous studies, it was determined that the 10k DPPH has a higher antioxidant capacity, but due to the low molecular weight fucoidan, there is a lower sulfate content. Therefore, it is speculated that the reducing power of fucoidan is also related to the sulfate content.

## Conclusion

Fucoidan and its fractions extracted from Weihai *U. pinnatifida* have good antioxidant capacity. The results show that the low molecular weight fucoidan fraction contains lower sulfate and uronic acid content compared with the high molecular weight fraction and the crude fucoidan. However, it still possesses a strong primary antioxidant activity. The possible explanation is that the low molecular weight fraction may contain oligosaccharides, phenolic compounds and pigment molecules. For the secondary antioxidant capacity, the 300k antioxidant capacity is stronger than 10k. On the other hand, the high molecular weight fucoidan fraction is higher than that of low molecular weight fucoidan in terms of iron reducing power. Based on FTIR and NMR analysis all fractions and crude fucoidan contain the characteristics of chemical bonds of fucoidan standard. It was confirmed that WH is proposed to be composed of an alternating (1 → 3)-_D_-galactose-(1 → 3)-_L_-fucose and (1 → 3)-_D_-galactose-(1 → 4)-_L_-fucose backbone. In conclusion, fucoidan extracted from Weihai *U. pinnatifida* possesses bioactivity, which makes it a valuable resource to be utilized in nutraceutical, pharmaceutical, and cosmeceutical industries.

## Data Availability Statement

The raw data supporting the conclusions of this article will be made available by the authors, without undue reservation.

## Author Contributions

JL and YZ: conceptualization, resources, and supervision. JY, QL, JW, XY, SY, WZ, and YL: methodology and formal analysis. WW, YZ, JY, QL, JW, XY, SY, WZ, and YL: validation. JY, QL, and JW: investigation. JY, QL, JW, and XY: data curation. JY, QL, AH, YZ, and JL: writing—original draft preparation. JY, WW, AH, YZ, and JL: writing—review and editing. JY and YZ: project administration. YZ: funding acquisition. All authors have read and agreed to the published version of the manuscript.

## Conflict of Interest

The authors declare that the research was conducted in the absence of any commercial or financial relationships that could be construed as a potential conflict of interest.
